# Case Report: Cerebral venous sinus thrombosis in the setting of iron deficiency anemia and a high level of lipoprotein (a) in a child

**DOI:** 10.3389/fped.2025.1449323

**Published:** 2025-03-10

**Authors:** Yomara Yarden Bustamante, Ulrike Seebeck, Martina Bührlen, David Overberg

**Affiliations:** ^1^ Pediatric Intensive Care, Eltern-Kind-Zentrum Prof. Hess, Klinikum Bremen Mitte, Bremen, Germany; ^2^ Coagulation Outpatient Clinic, Eltern-Kind-Zentrum Prof. Hess, Klinikum Bremen Mitte, Bremen, Germany

**Keywords:** iron deficiency anemia, lipoprotein (a), cerebral venous sinus thrombosis, cerebral thrombosis, thrombosis, case report, pediatric

## Abstract

Cerebral venous sinus thrombosis (CVST) is a rare but life-threatening condition among children. Several case reports have linked CVST formation to severe iron deficiency anemia (IDA). Iron deficiency anemia remains a public health problem, even in high-income countries. Among the thrombophilic factors accepted by some authors is an elevated lipoprotein (a). This is a case of a two-year-old girl with cerebral venous sinus thrombosis in the setting of IDA and high lipoprotein (a). These factors have been correlated with increased frequency in several clinical reports.

## 1 Introduction

IDA remains a prevalent disease worldwide (1, 2). The consequences of this disease are well known, especially in neurological development (3). Several case reports describing the correlation between iron deficiency anemia and CSVT are found in the literature (4, 5). CSVT is a critical condition in children whose etiology is varied (3). At the moment there are several hypotheses as to how these two clinical conditions may be linked (6–8). Additionally, in prothrombotic states, the role of lipoprotein (a) as an etiological factor is discussed and considered (9, 10). There are not many cases published with the relation between these factors. In this case report we present the case of a child with CSVT in the setting of IDA and high lipoprotein (a) as well as some of the literature reported with similar cases.

## 2 Case description

A two-year-old female arrived at the emergency department of a neighboring city hospital with history of lack of appetite, vomiting, fever, headache, and fatigue. She was previously healthy. Symptoms started three days before the consultation. Her general condition worsened, she stopped speaking and remained somnolent. Based on the clinical presentation and a moderately elevated spinal fluid protein (162.9 mg/dl), encephalitis was suspected. Therefore, she was treated with acyclovir until herpes-induced encephalitis was ruled out with a Herpes PCR-test. During admission, she presented repetitive episodes of disturbed consciousness, managed as seizures. MRI of the brain demonstrated cerebral venous sinus thrombosis (CVST). She was referred to our hospital for further evaluation and treatment.

The patient was then admitted into the pediatric intensive care unit in stable condition. The vital signs at her admission were heart rate 120 per minute, breathing rate 30 per minute, blood pressure 120/70 mmHg, and temperature 37.2°C. She weighed 12 kg (48 percentile). The patient was somnolent, showing selective stimulus defense and partial spontaneous movements in the physical examination. The Glasgow coma score was 9–10 points. The movements of the neck were restrictive, showing meningism signs. Muscle reflexes could be triggered on both sides and were normal. Muscle tone and strength were reduced. The Babinski reflex was negative on both sides. The pupils were equal, round, and reactive to light. The examination of the ears, nose, and throat was normal; there was no evidence of sinusitis or mastoiditis. The remaining physical examination was normal. Her mucosa and skin looked pale.

The parents reported neither family history of thrombosis nor strokes.

## 3 Diagnostic assessment

The laboratory chemistry parameters revealed a marked IDA with hemoglobin of 6.1 g/dl (10.8–12.8 g/dl), MCH 14.4 pg (23–31 pg), MCV 52.6 fL (73–101 fL), platelets 832/nL (140–360/nL.), ferritin 4.3 μg/L (6–60 μg/L), transferrin 3.06 g/L (1.9–3.0 g/L), transferrin saturation 2% (16%–45%). The blood smear showed a pronounced iron deficiency. There was no evidence of additional hemoglobinopathy (hemoglobin A 97%, hemoglobin A2 2.4%, hemoglobin F 0.6%, hemoglobin S, C, D and E were not detected).

**Figure 1:**
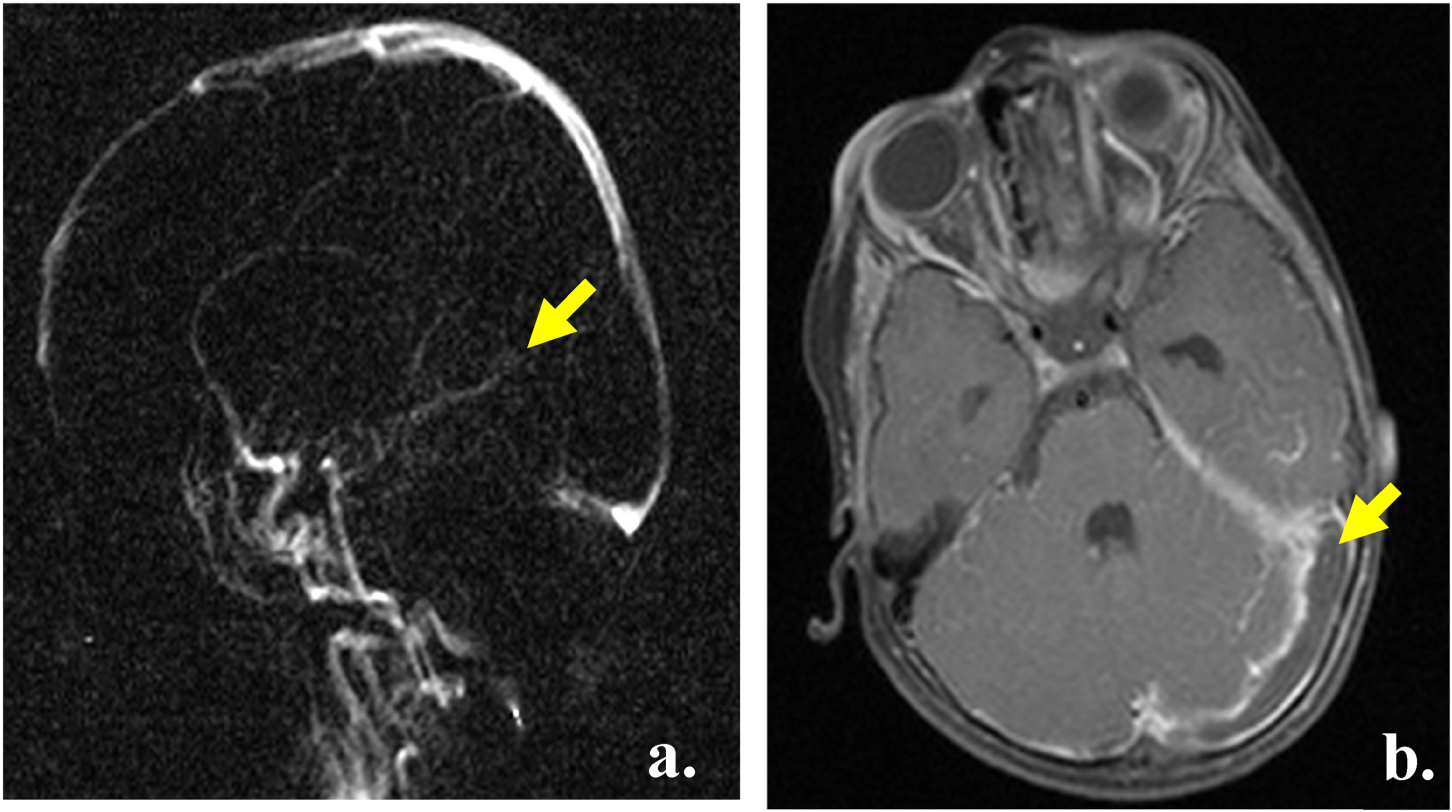
Radiological diagnosis. **(a)** MRI head, TOF venogram, yellow arrow showing the absence of flow in the sinus rectus and cerebral veins. **(b)** MRI head, T1, yellow arrow showing the thrombus extending into the left transverse sinus.

MRI and MRA showed an occlusive thrombus involving the rectus sinus, internal cerebral veins (Figure 1a) and left transverse sinus (Figure 1b). The brain parenchyma was unremarkable with no evidence of stroke or hemorrhage.

Our patient received a red blood cell transfusion. Additionally, she was treated with intravenous iron, and an oral iron substitution therapy was begun. The follow-up showed an adequeate improvement in the hemoglobin level. Therapeutic enoxaparin (1 mg/kg/dose twice daily) was administered. A significant improvement of neurological symptoms was seen within two days. The dose was regulated according to the anti-factor-Xa-activity. After one week, we transferred her to a pediatric ward with almost full neurological recovery.

During her admission, a detailed history revealed an insufficient and unbalanced oral food intake, mainly based on cow's milk. We initially began giving calorically enriched sip feeds and advised the family of the need for whole-food nutrition.

The patient was released from the hospital in a significantly improved general condition. The hemoglobin level was 9.5 g/dl, and the platelets were 546/nL. In a control 3 months after discharge, platelet values were in the normal range. A detailed thrombophilia workup revealed an increased lipoprotein (a) up to 86 mg/dl (0–29.9 mg/dl) as well as a transient detection of low-titer anticardiolipin Ig- G antibody. All other thrombophilia risk markers including protein C activity, protein S activity, antithrombin activity, factor V Leiden mutation, prothrombin mutation G20210 A, lupus anticoagulant, beta-2-glycoprotein-antibodies, anticardiolipin IgM and IgA-antibodies were negative. Factor VIII activity and von Willebrand factor parameters also showed normal results.

**Figure 2:**
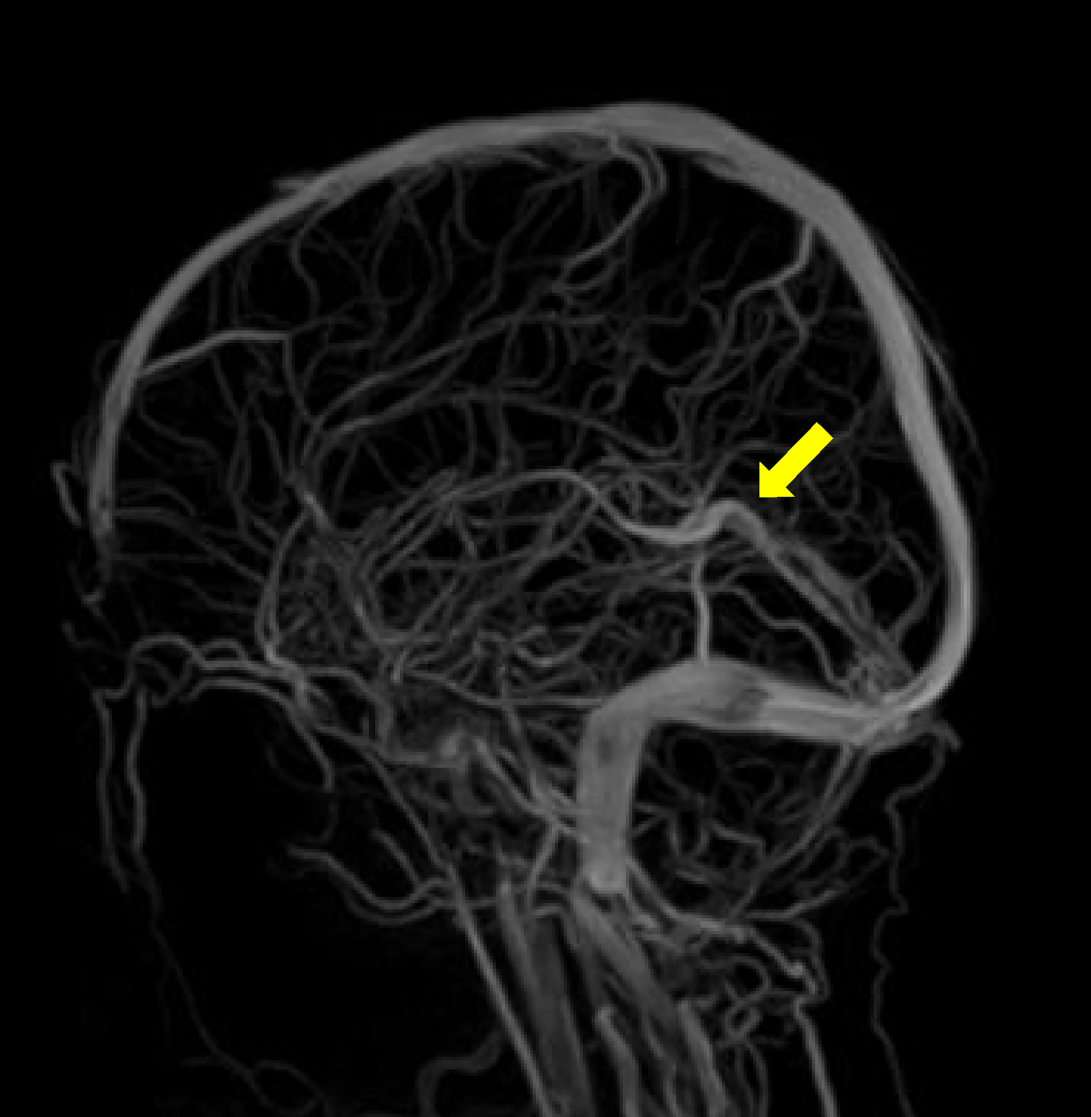
Two months follow up MRI head, TOF venogram, the arrow shows reestablished flow in the sinus rectus and cerebral veins.

**Figure 3:**
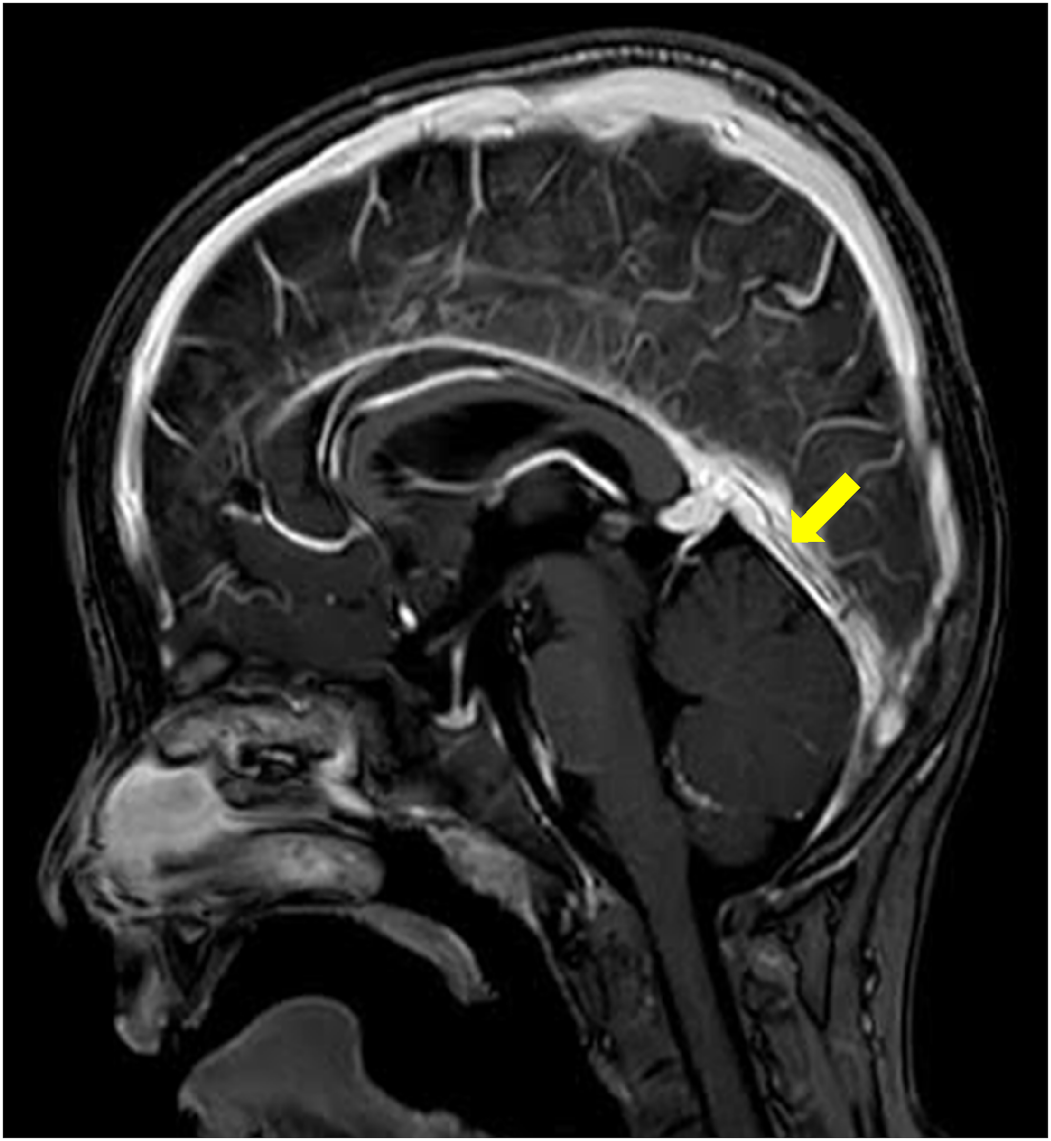
One-year follow-up. MRI of the brain after contrast administration demonstrates the presence of T1 hypointense linear filling defects within the straight sinus (arrows) in keeping with chronic non-occlusive thrombi.

Two months later, the MRI follow up showed a marked improvement (Figure 2), which correlated well with the clinical condition. One year later, there were still residual wall thrombi in the rectus sinus and the left transverse venous sinus up to the left jugular vein (Figure 3). The previously occluded internal cerebral veins were recanalized. Due to the extensive thrombosis, the absence of an external risk factor and the presence of two mild to moderate endogenous thrombophilia risk factors, the anticoagulation was continued for one year. The hemoglobin, ferritin, transferrin, and transferrin saturation were normalized. The patient recovered well according to the information from the parents and pediatrician.

## 4 Discussion

Cerebral venous sinus thrombosis (CVST) is a pediatric stroke syndrome (11). The clinical manifestations of CVST vary across age groups and include, like our patient, headache, nausea, vomiting, seizures, and altered mental status (11).

CVST causes symptoms and signs by two mechanisms: thrombosis of the cerebral veins and thrombosis of the major sinuses. In the majority of patients, these two processes co-occur (3). Thrombosis within the venous system results in outflow obstruction, venous congestion, and a consequent increase in capillary hydrostatic pressure, producing edema (12).

CVST has a comprehensive and often multifactorial pathophysiology. Among the causes, several case reports have linked severe IDA to CVST formation (4, 5, 13–15). However, many of these studies are small series of cases. CVST is not a common pathology in children. Therefore, many conclusions and clinical data result from adult case reports.

Our patient's treatment with low molecular weight heparin was successful, as shown in the MRI made months after treatment. At the time of the diagnosis of our patient, clinical trials were lacking in pediatric CVST. Therefore, neonates and children received anticoagulant therapy based on adult studies. This therapy aims to restore flow and prevent the further spread of thrombus (1, 16). Anticoagulation appears to be safely used in pediatric CVST. On the other hand, non-treatment is associated with thrombus propagation in 25%–33% of cases (11, 17). In 2021, the direct oral anticoagulant rivaroxaban was licensed for the treatment of childhood thrombosis. The substudy of the EINSTEIN-Jr Approval Study randomized 114 children with CVT and showed the safety and efficacy of rivaroxaban and standard anticoagulants (18).

Anemia is a global health problem with a prevalence of 32.9% among the population, in which IDA is the most common cause (2). Among all children under 5 years of age worldwide, approximately 40% are known to be affected by IDA (1). In 1989 a case report suggested IDA as a possible cause of CVST in an otherwise healthy 22-months-old boy (15). Three case reports with patients between 9 and 27 months old having CVST postulate the concomitant IDA as probable cause. Further evaluation showed no prothrombotic disorders in these cases (5, 13).

In a 2005 publication, 42 children with CVST were studied (6). In 50% of the cases, anemia with or without microcytosis (probable iron deficiency) was present, which is as common as prothrombotic disorders. A study in Canada compared 15 children with stroke vs. 143 healthy subjects in which IDA was found in more than 50% of all strokes (7). In 2015, a study in the Netherlands included 152 patients with a thrombotic event and 2,196 as control subjects (19). Anemia was present in 27% of CVST cases, and the association was stronger in patients with microcytic anemia. In 2018, a publication summarizing the literature from 1972 to 2016 reported 54 arterial and venous thrombosis cases associated with IDA (8). Of these cases, three-quarters represented cerebral thrombosis. The reported cases of children were all cerebral venous occlusions. A case-control study in 2016 involving 21 stroke cases in Egypt showed that children who developed a stroke are 3.8 times more likely to have IDA (20). The study also determined a strong relationship between anemia and thrombocytosis among cases.

Three physiological mechanisms explaining IDA leading to stroke include a hypercoagulable state, thrombocytosis, and anemic hypoxia (21). IDA can contribute to a hypercoagulable state, predisposing children to develop CVST. In this type of anemia, the red cells have decreased deformability, causing a turbulent flow (16). This can increase blood viscosity and lead to activation of the coagulation cascade. Additionally, anemia increases cerebral blood flow due to the lower oxygen-carrying capacity. As a result of the increased blood velocity, turbulence might result in endothelial damage and inflammation, activation of the coagulation cascade, and the formation of thrombi (1, 13). This augmented cerebral blood flow was measured with Doppler sonography in 63 percent of studied children with IDA (22).

In addition, most cases report anemia with thrombocytosis. A well-known phenomenon is that anemia's high compensatory erythropoietin levels also induce reactive thrombocytosis. However, abnormal platelet activation and function are more important than absolute platelet count. Some cases of IDA and CVST have been reported without important thrombocytosis (23). Our patient had a maximal number of platelets of 832/nL.

Iron deficiency can also promote reactive thrombosis *per se* (24). Iron is also involved in regulating platelet levels, inhibiting its production (13, 15). A study in animal models showed more significant growth and dimensions of venous thrombi in iron deficiency states (25). This study also confirmed that iron deficiency induces thrombocytosis and increases thrombus size. A different mouse study demonstrated that a lack of iron led to a bend towards megakaryocyte progenitors and subsequent thrombopoiesis at the expense of red blood cells (26).

Potaczek et al. (27) showed an association between iron deficiency and an increased venous thromboembolism (VTE) recurrence rate. They proposed that iron deficiency, and not anemia itself, might be the leading risk factor among the adult population with VTE in their study. The mechanisms that could explain this relationship are reduced antioxidant defense due to iron deficiency and reduced activity of glutathione peroxidase. These reductions correspond to an increased platelet aggregation induced both by collagen and ADP and reactive thrombocytosis (27). Usually, the cases reported in the literature are relating IDA, thrombocytosis, and venous thrombosis. However, a case report of an adult has been published documenting the association of IDA, thrombocytosis, and arterial thrombosis (28). There are several other studies relating IDA to ischemic cerebrovascular accident (23).

The metabolic demand at the tissue level rises under stress or infections. This can create anemic hypoxia and can predispose to venous thrombosis (29). High levels of C-reactive protein are also associated with the formation of dense fibrin networks less susceptible to lysis (27).

Our patient was fed only cow milk, which could explain the possible etiology of food-related anemia. Multiple reports describe cow milk consumption as a cause of IDA, as seen in our patient. A diet consisting mainly of cow milk leads to iron deficiency due to its low iron content. Likewise, the high calcium and casein in cow milk also inhibit the absorption of non-heme iron, worsening anemia. Another mechanism can be occult intestinal bleeding associated with cow milk consumption (1, 13, 30).

The severe IDA might have played an essential role in our reported case's emergence of sinus venous thrombosis. As mentioned before, the presence of IDA can predispose to a thrombotic state. Her follow-up in the coagulation outpatient department provided additional information about her underlying congenital thrombophilia risk factor high lipoprotein (a). It is still widely discussed whether the high lipoprotein (a) level is directly associated with thrombotic events. The lipoprotein (a) has been described to have thrombogenic properties, including the inhibition of fibrinolysis, induction of plasminogen activator inhibitor type 1, and increase of the tissue factor pathway inhibitor activity and platelet responsiveness. Studies show the association of lipoprotein(a) and other thrombophilic risk factors with the presence and recurrent incidence of VTE in children and adolescents (31). However, a meta-analysis of six studies among 1,155 young patients did not find any significant association between high lipoprotein (a) and recurrent VTE. Elevated lipoprotein(a) *per se* did not increase the risk. However, the combination of lipoprotein(a) levels higher than 30 mg/dl with at least one other thrombophilic risk factor increased the risk (31).

A Turkish study published in 2021 included 29 children with CVST (32). Among this group, 26 children had an acquired or/and an inherited thrombophilic risk factor. Seven of the 21 patients had acquired thrombophilic risk factors, such as dehydration and infection, and also had inherited thrombophilic risk factors. The two most common inherited thrombophilic risk factors were factor V Leiden mutation and elevated lipoprotein (a) higher than 30 mg/dl. Skuza, et al (9). reported in a study among 80 adult patients with CVST that a high level of lipoprotein (a) is associated with decreased clot permeation. A Danish study with more than 2,000 cases of VTE showed that extremely high levels of lipoprotein (a) 100 mg/dl compared with low levels of 5 mg/dl carry a hazard ratio of 1.33 for thromboembolism (33). High levels of this lipoprotein likely lead to a higher risk of VTE. However, it seems more directly related to myocardial infarction and aortic valve stenosis (33).

As in the case of our patient, there is a case report of a 21-year-old woman having CVST with absence of a thrombophilia family history but positive results for IDA, and a high level of lipoprotein (a) by 100 mg/dl (10). Our patient had a lipoprotein (a) value (86 mg/dl) three-fold the reported risk level. This condition and the severe IDA might have predisposed to CVST. However, as shown in the literature, more consensus and further studies are required.

Over 1.2 billion people are affected by IDA worldwide (25). Most studies are done in adults whose physiopathology and comorbidities differ widely from the children. Some factors still need to be clarified, such as why some patients develop CVST, and others do not while presenting the same level of iron deficiency.

In conclusion, there seems to be a correlation between IDA and CVST. Further studies must be made to clarify and understand the exact relation between these conditions. Meanwhile, prevention and early treatment of anemia in children and populations with chronic diseases is a valuable measure. Primary prevention of IDA needs to be *a priori*ty for neurodevelopment and for avoiding severe complications like CVST. The strict treatment and follow up of IDA as well as the prevention by the education of parents with nutritional facts must continue to be more prevalent.

## Data Availability

The original contributions presented in the study are included in the article/Supplementary Material, further inquiries can be directed to the corresponding author.
